# Inferring ion channel densities from spike data

**DOI:** 10.1186/1471-2202-12-S1-P12

**Published:** 2011-07-18

**Authors:** Oliver Muthmann, Matthias H Hennig, Upinder S Bhalla

**Affiliations:** 1 Department, National Centre for Biological Sciences, Tata Institute of Fundamental Research, Bangalore, 560065, India; 2 Institute for Adaptive and Neural Computation, School of Informatics, University of Edinburgh, Edinburgh, EH8 9AB, UK

## 

In high density multiple electrode array recordings, it is now possible to simultaneously measure spiking activity of a large number of single neurons in cultures. However, the connectivity and synaptic weights are not accessible, which makes it difficult to make a good comparison with simulated data. In fact, the spike pattern of a neuron strongly depends on its input, whereas the effect of changes in ion channel densities often makes only a small difference. Thus, to infer neuronal properties from such data, one needs to take into account its synaptic inputs.

We stimulated a reduced neuron model based on the model of [1] with different Poisson spike trains and randomly permuted the synaptic weights, keeping the sum of weights constant. We then locally compared interspike interval (ISI) sequences between pairs of the resulting spike trains. This was done by ranking ISIs according to their lengths around different points in time, calculating the difference of those ranks and summing up the absolute value of the difference vector. We chose to compare ranks instead of absolute values as they depend less on the firing rate of the neuron. As a reference we used a perfect (PIF) and a leaky integrator (LIF) fed by shared Poisson input. We find that

average rank differences can be matched by the LIF model when choosing an appropriate time constant. As shown in Fig. 1, strongly overlapping inputs are needed to produce similar firing patterns, unlike in the case of the perfect integrator.

**Figure 1:**
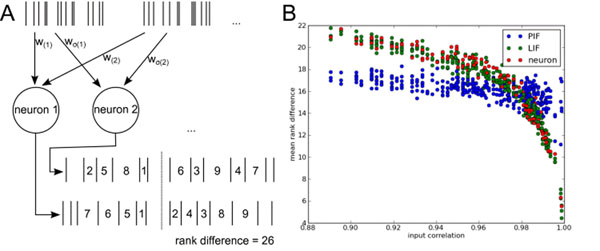
**A** Sketch of the setup. Every neuron is stimulated by the same set of Poisson inputs using randomly permuted weights. Pairs of spike trains produced by the neurons are then compared by calculating the difference of local rank vectors. The interval at the reference time is omitted from the analysis since it would induce a bias. **B** Result for the reduced neuron model (pooled data from 3 sets of input weights).

Having an estimate on how much a different connectivity perturbs measured spike trains, we will further investigate whether changes in ion channel densities can be detected by monitoring the activity of upstream neurons and the neural spiking.

## References

[B1] Poirazi P Brannon T Mel BW Arithmetic of subthreshold synaptic summation in a model CA1 pyramidal cell Neuron 2003 37 977 987 10.1016/S0896-6273(03)00148-X 12670426

